# Continuous blood exchange in rats as a novel approach for experimental investigation

**DOI:** 10.1038/s41598-024-63049-0

**Published:** 2024-05-28

**Authors:** Siya Pei, Yanjie Wang, Zhimin Zhang, Cheng Mei, Wenyu Yin, Xiangjie Fu, Danyang Yan, Yuanyuan Zhu, Tianli Lin, Yiran Zhou, Ning Li

**Affiliations:** 1 Department of Blood Transfusion, Xiangya Hospital, Central South University, 87 Xiangya Road, Changsha, 410008 Hunan Province China; 2 Key Laboratory of Viral Hepatitis of Hunan Province, Xiangya Hospital, Central South University, 87 Xiangya Road, Changsha, 410008 Hunan Province China; 3 National Clinical Research Centre for Geriatric Disorders, Xiangya Hospital, Central South University, 87 Xiangya Road, Changsha, 410008 Hunan Province China

**Keywords:** Experimental models of disease, Haematopoietic system, Biological techniques

## Abstract

Blood exchange therapy, specifically Whole blood exchange (WBE), is increasingly being utilized in clinical settings to effectively treat a range of diseases. Consequently, there is an urgent requirement to establish convenient and clinically applicable animal models that can facilitate the exploration of blood exchange therapy mechanisms. Our study conducted continuous WBE in rats through femoral and tail vein catheterization using dual-directional syringe pumps. To demonstrate the applicability of continuous WBE, drug-induced hemolytic anemia (DIHA) was induced through phenylhydrazine hydrochloride (PHZ) injection. Notability, the rats of DIHA + WBE group all survived and recovered within the subsequent period. After the implementation of continuous WBE therapy day (Day 1), the DIHA + WBE group exhibited a statistically significant increase in red blood cells (RBC) (P = 0.0343) and hemoglobin (HGB) levels (P = 0.0090) compared to DIHA group. The rats in the DIHA + WBE group exhibited a faster recovery rate compared to the DIHA group, indicating the successful establishment of a continuous blood exchange protocol. This experimental approach demonstrates not just promising efficacy in the treatment of DIHA and offers a valuable tool for investigating the underlying mechanisms of blood exchange. Furthermore, it has a great potential to the advancement of biomedical research such as drug delivery exploration.

## Introduction

Currently, blood exchange therapy, including whole blood exchange (WBE), are gradually being widely applied in clinical practice for the treatment of various diseases such as autoimmune hemolytic anemia^1^, sickle cell anemia^2^ and other clinically relevant disorders, including but not limited to paroxysmal nocturnal hemoglobinuria (PNH)^3^, intoxication^4^, thrombotic thrombocytopenic purpura (TTP)^5^ with good therapeutic outcomes. Based on the modified blood exchange technique developed by Professor Li BJ, not only has this method achieved favorable clinical outcomes, but it has also been documented in several clinical articles^1,3,6^. A real-world study exploring the efficacy of WBE in Autoimmune Hemolytic Anemia Secondary to Systemic Lupus Erythematosus (SLE-AIHA) patients showed that this treatment may serve as a safe and beneficial alternative for refractory severe SLE-AIHA ^6^. Another study indicated that lymphoplasmapheresis, a novel blood exchange combining conventional plasma exchange and lymphocyte removal^7,8^, required less plasma and was significantly effective in treating patients with severe myasthenia gravis^7^. Despite the good clinical efficacy of blood exchange include plasma exchange (PE), its therapeutic mechanism remains unclear^9,10^. Therefore, there is a pressing need to establish convenient and clinically relevant animal models for exploring the mechanisms of blood exchange therapy, in order to facilitate our understanding of this treatment modality.

Blood exchange models are experimental methods that involve the transfer of blood or blood components between two animals, which are commonly used to investigate the effects of certain substances or treatments on the blood and other bodily systems^10–16^. Previous study show that those models have been used for a variety of purposes, including the study of blood disorders^11,12,17^, the investigation of immune system function^18^, and the testing of medical therapies^19–21^.

Due to their similar anatomical and physiological characteristics to humans, large animals such as dogs, and pigs were used in blood exchange models^12,14,22,23^. Those large animals are used in complex and discontinuous blood exchange methods, resulting in high experimental costs and time, leading to high failure rates^12,22^. Recent studies have explored alternative methods for blood exchange in animals, such as transfusion of small animals through intermittent infusion by the use of microcirculation electronic pumps^10,24^. Although these research methods^10,24^ have reduced experimental costs, simplified procedures, and increased experimental control compared to previous study^16^, they^10,24^ often require pre-implanted jugular veins, which can lead to serious surgical injuries. And those methods has limitations include intermittent plasma exchange^10^, a small exchange volume per time^25^.

Here, we developed a WBE method in rats, which involves continuous WBE with minimal incisions using easily accessible materials and subjects, ensuring effective blood volume exchange. This approach has demonstrated a low occurrence of postoperative infections and mortality rates, presenting an experimental avenue for delving into the potential mechanisms of blood exchange therapy. Supplementary Table 1 has been summarized to compare representative animal models for blood exchange, highlighting the efficiency of WBE method compared to the established methods^10,12–14,23^. By circumventing the need for jugular vein catheterization and discontinuous exchange protocols, this method mitigates the previously encountered surgical trauma and complications, thereby providing a reliable and less invasive alternative for long-term studies. The aim of our study is to enable reproducible, continuous blood exchange experiments using accessible consumables and rat models to address challenges in blood exchange models, offering a novel potential approach to investigate the efficacy mechanism of blood exchange on various disease conditions for intended study.

## Materials and methods

### Animals

This study was approved by the Central South University Experimental Animal Welfare Ethics Review Application and the Central South University Science Research Project Experimental Animal Welfare Ethics Approval (ethics approval number: CSU-2023-0065) and was conducted in compliance with all relevant regulations governing the ethical use of laboratory animals for research purposes. And the study adheres to ARRIVE Essential 10 of the ARRIVE guidelines 2.0^26^.

In this study, Healthy adult male Sprague-Dawley (SD) rats weighing between 290 and 340 g, were sourced from China Charles River Laboratories. Prior to use in the study, the rats were screened and certified to be free of specific pathogens. These rats were housed in the Experimental Animal Department of Central South University, where they were maintained in a controlled environment with a 12-h light/12-h dark cycle and provided with ad libitum access to food and water, both of which were sourced from the same department. The rats were accommodated in pathogen-free cages, specifically designed for animal experiments, with each cage housing three rats. After a 1-week acclimation period, during which their health was closely monitored, the rats were randomly assigned to their respective experimental groups.

### Materials

Isoflurane was administered through a precision vaporizer to achieve proper anesthesia. Povidone-iodine was used for skin preparation before surgery. Heparin was used to prevent blood clotting during experimental procedures. 0.9% sodium chloride injection was used for fluid replacement and electrolyte balance maintenance during the experiments. All solutions were obtained from reliable medical experimental procedure suppliers and prepared according to standard protocols. All required materials are summarized in Table 1.

**Table 1:** Required materials.

Materials	Manufacturers
**Reagents**	
Isoflurane	RWD Life Science Co., Ltd Shenzhen China
Povidone-iodine	Mingde Co., Ltd Dezhou China
Heparin	MedChemExpress New Jersey USA (CAS-NO-9041-081)
0.9% Sodium chloride	CR Double-crane Co., Ltd Pingdingshan China
Phenylhydrazine hydrochloride	Sigma-Aldrich (CAS-NO-59-88-1)
**Equipment**	
R540IE Enhanced small animal anesthesia machine	RWD Life Science Co., Ltd Shenzhen China
DSC-B01/W150-B01Separable infusion pump	Baoding Acmer Precision Pump Co., Ltd Baoding China
Electronic weighing scale	Wuxinhengqi Co., LTD Jinhua China
Professional pet trimmer	Zhuochuang Co., Ltd Wenzhou China
Electronic timer	Baijie Co., LTD Huzhou China
**Surgical tools**	
Shadowless surgical lamp	Medilan Medical Equipment Co., LTD Nanjing China
Fixation plate	Zhongkehuida Co., LTD Beijing China
Hemostatic forceps	ShangHai Medical instrument Co., LTD Shanghai China
Surgical scissors	ShangHai Medical instrument Co., LTD Shanghai China
Ophthalmic forceps	ShangHai Medical instrument Co., LTD Shanghai China
Animal dissection auxiliary retractor	Dasijiaer Co., LTD Huaibei China
Micro Vannas scissors	Dasijiaer Co., LTD Huaibei China
Vascular clamp	Dasijiaer Co., LTD Huaibei China
Handle for surgical blade	Dasijiaer Co., LTD Huaibei China
Sterile plastic handle surgical blade 36#/S4	Fu Yang Medical Suture Neadle factory, Hangzhou China
**Related equipment**	
Disposable sterile syringe 5 mL	Guangzhou Jet Bio-Filtration Co., Ltd Guangzhou China
Disposable centrifuge tube 15 mL	Guangzhou Jet Bio-Filtration Co., Ltd Guangzhou China
Disposable centrifuge tube 50 mL	Guangzhou Jet Bio-Filtration Co., Ltd Guangzhou China
Single-use venous blood collection container	Hunan sanli Co., Ltd Liuyang China
Absorbable surgical suture 5–0	Jinhuan Medical Co., LTD Shanghai China
Single-use three-way connector	Jiangsu Huaxing Medical Devices Industry Co., Ltd. Yangzhou China
Sterile Acrodisc® leukocyte filter needle 5 MM	PALL Corporation New York USA
Disposable indwelling needle 24G	Jiangxi Huali Medical Instrument Co., Ltd. Ganzhou China
Disposable catheter 26T	Dasijiaer Co., LTD Huaibei China

### Experimental design

**Figure 1:**
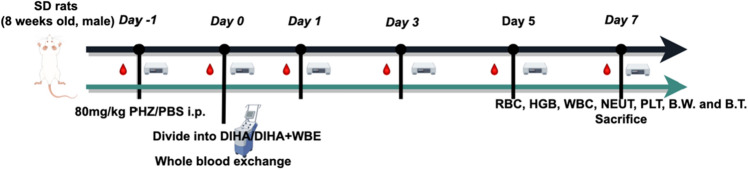
Schematic demonstrating the experimental timeline. Sprague-Dawley (SD) rats, Phenylhydrazine hydrochloride (PHZ), Phosphate buffered saline (PBS), Intraperitoneal injections (i.p.), Whole blood exchange (WBE), Red blood cells (RBC), Hemoglobin (HGB), White blood cells (WBC), Neutrophils (NEUT), Platelets (PLT), Body temperature (B.T.) and Body weight (B.W.)

To demonstrate the applicability of continuous WBE, we established the drug-induced hemolytic anemia (DIHA) model and applied it to our experimental scenario. The current study employed the minimum number of animals (each group n = 3) necessary for statistical analysis, thereby minimizing the impact on experimental animal welfare. A total of 12 SD rats were utilized in this study. Among them, 3 rats served as the healthy blood donor group, while the remaining 9 rats underwent the following data monitoring. The 9 rats were randomly allocated into three groups: the healthy control group, the DIHA + WBE group, and the DIHA model group. The DIHA model was induced by administering intraperitoneal injections (i.p.) of Hydrazine hydrochloride (PHZ) 80 mg/kg to all groups^27,28^, except for the healthy control group. Blood samples were collected at consistent time points of Day − 1, Day 0, Day 1, Day 3, Day 5 and Day 7 by experienced and designated experimenters. Subsequently, all samples were promptly transferred to Xiangya Hospital's transfusion department for analysis using the Mindray CL-600 blood routine analyzer, aiming to minimize data variability. Following the experimental timeline outlined in Fig. 1.

## Main steps of the study

### Reagent preparation

Prepare the required reagents for the experiment prior to commencement: prepare heparin at a concentration of 30 IU/mL using physiological saline and set it aside; prepare iodine solution and physiological saline (Reagents, Table 1).

*Caution!* All reagent preparation procedures should be conducted in a sterile laminar flow hood to prevent contamination.

### Catheter and pump preparation

Using the prepared 30 IU/mL heparin pre-soak the disposable rat arterial and venous catheters, connecting tubes, three-way stopcocks, and sterile medication dispensers, and arrange them in a sterile surgical field (Surgical tools, Table 1). Record the volume of heparin used for pre-soaking and ensure that the air bubbles are removed from the catheters, as their presence may cause harm to the experimental animals.

*Caution!* All catheters should be prepared on a sterile work surface.

### Transfusion blood preparation

Preparation of the infusion fluid required for the study before commencing the formal experiment. The blood of healthy rats was utilized as the source of infusion whole blood, obtained via cardiac puncture under general anesthesia in this experiment. Anticoagulation was achieved by heparinization with heparin 0.024 IU/ml^29,30^. The required volume of the infusion fluid is calculated using the commonly used approximate formula for rat blood volume, where total blood volume^31^ (ml) ≈ body weight (g) × 0.06. It is noteworthy that a preliminary cross-matching process utilizing a fully matched allogeneic donor is imperative to avoid potential hemolytic reactions during the experiment and ensure its success. The cassette microcolumn gel test involved the addition of 10 μL 4% donor RBC and 40 μL recipient plasma in the forward typing, and 10 μL 4% recipient RBC and 40 μL donor plasma in the reverse typing. Following incubation at 37 °C for 10 min, centrifugation for 5 min, and interpretation of results, the distribution of RBC was observed. Negative results were indicated by RBC at the bottom of the microcolumn, while positive results were indicated by RBC bound within or above the microcolumn of the gel.

*Caution!* Ensure a successful cross-matching process with age-matched donors.

### Anesthesia, medication, and animal preparation

The experimental rats were anesthetized with an anesthesia machine (Equipment, Table 1), then expeditiously positioned in a supine stance on a surgical table, and the depth of anesthesia was assessed. The groin region was disinfected with iodine and shielded with sterile drapes. Following an incision in the groin area, the femoral artery, along with the femoral vein and nerve, was exposed. The femoral artery was dissected bluntly, and two absorbable surgical sutures were placed on the femoral artery without knotting. The distal end was ligated with a surgical suture, a clamping vascular clip attached to the proximal end. A V-shaped incision was carefully created using microscissors. Subsequently, a disposable arterial catheter for rats was inserted through the incision and secured in place^32^. The femoral artery catheterization procedure was completed by covering the incision site with sterile gauze soaked in physiological saline solution. The other end of the catheter was connected to a sterile infusion set, which was mounted on a separate syringe pump. (Equipment, Table 1).

The tail end of the rats was disinfected with iodine, and the area 1/3 from the end was selected for cannulation and fixation of the tail vein using a disposable 24G arterial and venous indwelling needle. The other end of the indwelling needle was connected to a three-way connector or infusion set (depending on the experimental purpose) containing whole blood/blood components, which was fixed on another syringe pump. (Related equipment, Table 1).

**Figure 2:**
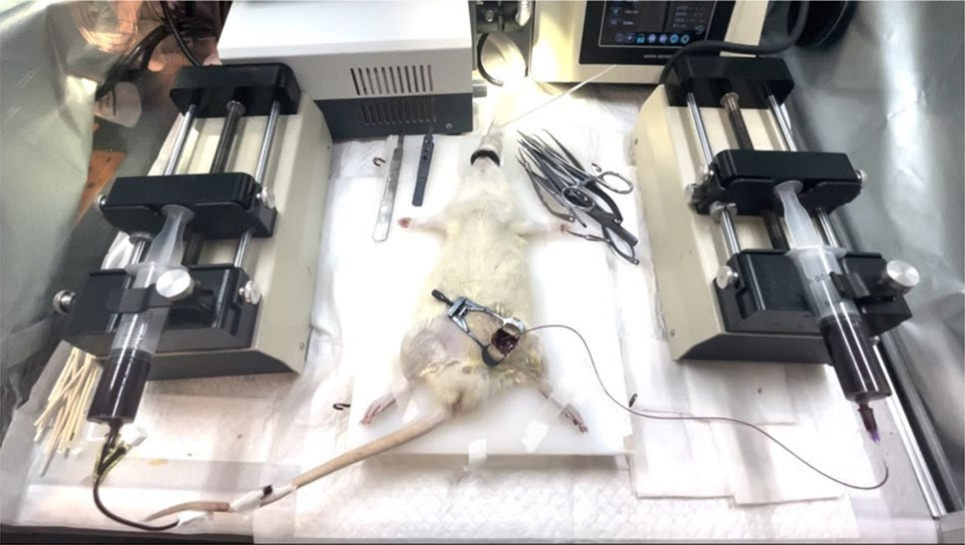
The overall view of the WBE procedure in rats.

Upon successful placement, the catheter is connected to the appropriate equipment and the exchange device is activated. The device connection for blood exchange was accomplished and the flow rate for blood collection/ infusion was established (Fig. 2).

*Caution!* The catheter is meticulously inserted into either the femoral artery or tail vein of the rat with great technical dexterity and precision to minimize any potential discomfort or harm to the animal.

Throughout the procedure, close attention is paid to the time, blood flow rate, velocity, and volume of the exchange, all of which are meticulously monitored and recorded to ensure precise data collection and analysis. Once the circulatory volume blood exchange is achieved, the catheter is carefully removed, the femoral artery is ligated, and the incision site is thoroughly irrigated with saline following iodine disinfection. The incision is then sutured, and the anesthesia machine is turned off, allowing the rat to awaken gradually over a period of 5–20 min. The entire continuous blood exchange process lasts for approximately 70–100 min examination (Supplementary Video).

*Caution!* It is crucial to maintain equivalent collection and infusion rates^33^ (200–300 µL/min) to ensure a balanced fluid equilibrium in rats, and prevent the occurrence of severe pathological injuries, such as shock or heart failure, that may lead to experimental failure. Special attention must be paid to maintaining a steady balance of fluid inflow and outflow to prevent any potentially detrimental effects.

### Postoperative management

After completion of the continuous blood exchange at Day 0, the surgical site was meticulously closed and sterilized. The rat was then allowed to recover consciousness and was subjected to routine prophylactic antibiotic therapy to prevent infection. To facilitate a rapid postoperative recovery, a warming pad was utilized after WBE at Day 0 for 4–6 h. Upon completion of the experiment at Day 7, the rats were subjected to euthanasia using a controlled carbon dioxide displacement rate of 50% volume per minute.

### Statistical analysis

Statistical analysis was conducted using GraphPad Prism version 9.4.1. The Shapiro–Wilk test was initially employed to assess data normality, considering the relatively small sample size across three distinct groups and multiple time points. Data transformation techniques were utilized to ensure adherence to normality assumptions if deviations were observed. Subsequently, tests for homogeneity of variances were performed, with the application of the Geisser-Greenhouse correction for unequal variances (P > 0.05). A repeated measures (RM) two-way ANOVA, incorporating Geisser-Greenhouse correction, was then carried out to accommodate matched values. Tukey's multiple comparisons test was utilized for post-hoc analyses, with individual variances calculated for each comparison. Data analysts were blinded to experimental groups. The results are presented as mean ± standard deviation (SD). A P-value, as determined by Tukey's multiple comparisons test, which less than 0.05 (*) was considered statistically significant.

## Results

### Establishment of the drug-induced hemolytic model and baseline parameter assessment

**Figure 3:**
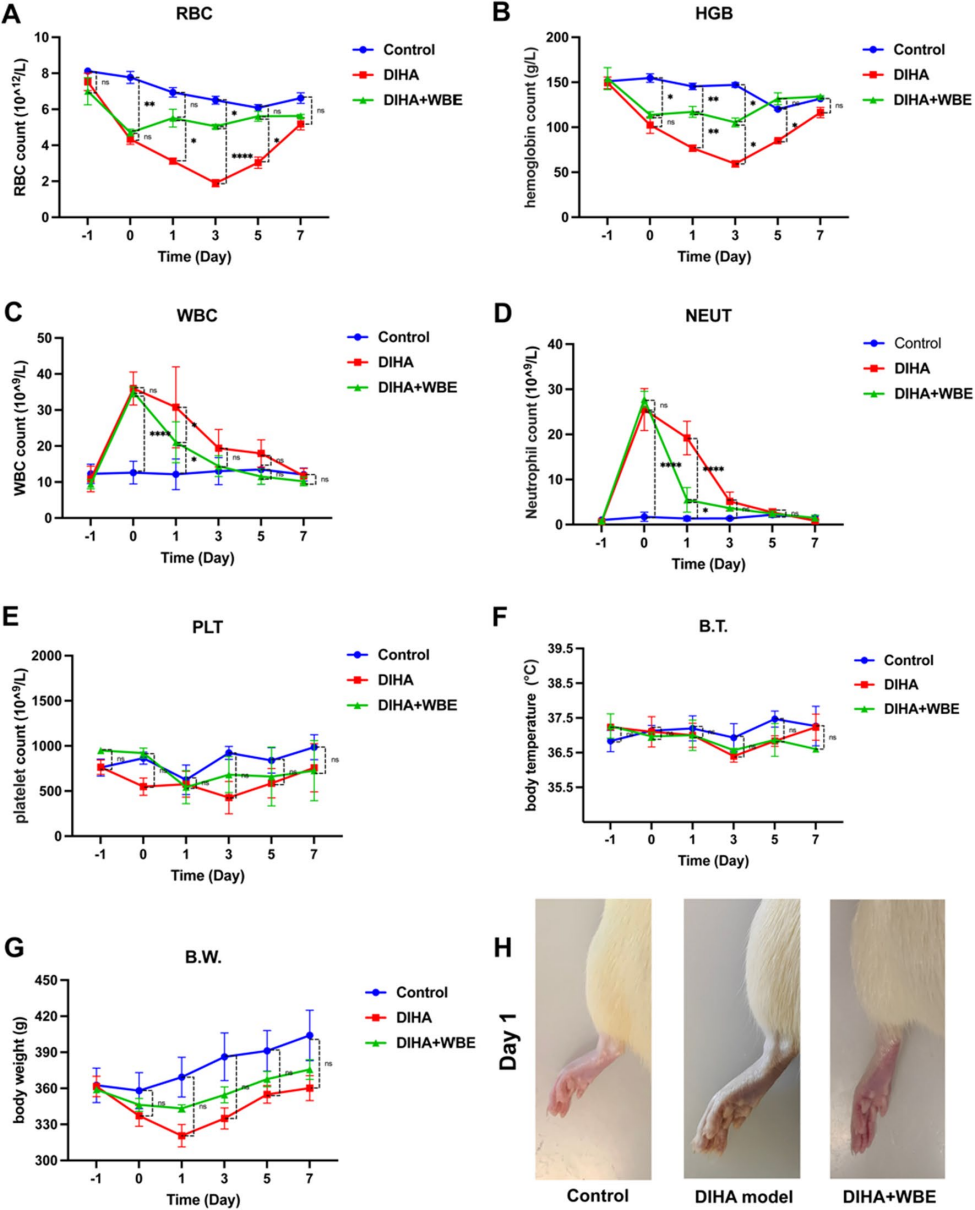
Efficacy of Continuous WBE Therapy in DIHA Rats. Assessment of RBC (**A**), HGB (**B**), WBC (**C**), NEUT (**D**), PLT (**E**), B.T. (**F**) and B.W. (**G**) before and after experimental procedure (each group n = 3). Photographs of the left hind paws of rats (**H**). Statistical analysis (*P* > 0.05, not significant, ns), asterisks denote significant differences, with *P ≤ 0.05, **P ≤ 0.01 and ****P ≤ 0.0001.

On the day preceding the commencement of the WBE experiment (Day − 1), retro-orbital venous blood samples were collected from all rats to assess baseline values of red blood cells (RBC) count, hemoglobin (HGB), white blood cells (WBC) count, neutrophil (NEUT), platelet (PLT). Clinical signs, including body weight (B.W.) and body temperature (B.T.) were measured. All the parameter data, presented as mean ± SD, has been documented in Supplementary Table 2 for reference in this study. The results demonstrated no statistically significant differences in the levels of RBC, HGB, WBC, NEUT, PLT, B.W. and B.T. among the three groups (Fig. 3A–H). Subsequently on Day 0, a drug-induced hemolytic model was established by administering intraperitoneal injections of PHZ to all groups except for the healthy control group. The data depicted in Fig. 3 on Day 0 exhibited a marked reduction in RBC count (P = 0.0189, P = 0.0029) and HGB levels (P = 0.0143, P = 0.0139), accompanied by a substantial increase in WBC count (P < 0.0001, P < 0.0001) and NEUT count (P < 0.0001, P < 0.0001) compared to the healthy control group, indicating the successful establishment of the drug-induced hemolytic model (Fig. 3A–D).

### Effects of WBE therapy on hematological parameters

After the application of continuous WBE therapy, we conducted assessments on Day 1 and observed the DIHA + WBE group has a significant increase in RBC (P = 0.0343), and HGB levels (P = 0.0090), accompanied by a noteworthy decrease in WBC (P = 0.0013) and NEUT count (P < 0.0001) compared with DIHA model group. Moreover, a visual assessment of the rat's paws on Day 1 demonstrated a discernible difference in coloration, the paws of the DIHA + WBE group exhibited a more vibrant and ruddy appearance, whereas the DIHA group displayed a pallid, grayish discoloration (Fig. 3H). On Day 3 post-treatment, the results of DIHA + WBE group still revealed a significant elevation in RBC (P < 0.0001) and HGB levels (P = 0.0122) compared to the DIHA group. Even though the results of the DIHA group raises gradually in RBC and HGB levels it remained significantly lower in RBC (P = 0.0131) and HGB levels (P = 0.0155) compared to the DIHA + WBE group on day 5 post-treatment. On the day 7 post-treatment, both of RBC and HGB levels showed no significant difference between all groups. Meanwhile, WBC and NEUT count are no significant difference between groups on Day 3 to Day 7 post-treatment. Concomitantly, we conducted evaluations on the PLT, B.W. and B.T. of the rats to assess the therapeutic efficacy of WBE intervention. Our results demonstrated no statistically significant variations in PLT, B.T. or B.W. between the experimental groups (Fig. 3E–G). These findings collectively demonstrate the beneficial impact of WBE therapy in ameliorating anemia in DIHA rats. Additionally, it is noteworthy that the implementation of WBE therapy exhibited no evidence of infection or discernible effects on coagulation function, B.W. or B.T in the rat model.

The rats treated with WBE exhibited immediate signs of alertness and liveliness upon awakening from anesthesia. They demonstrated independent feeding ability, and the wounds resulting from experimental procedure exhibited mild clotting and exudation on the day, with evident improvement in wound healing over time.

## Discussion

In current animal research methods for blood exchange, jugular vein catheterization is extensively utilized^11,24,34^. However, this highly invasive procedure may lead to significant injuries and uncertainties during experimentation (e.g., poor healing of the large wounds, difficulty in removing the catheter). Here, we summarized Supplementary Table 1^10,12–14,23^ that include representative animal models for blood exchange procedures, elucidating the efficacy, and potential benefits of our WBE procedure. Our WBE method, utilizing the femoral artery-tail vein exchange in rats, offers a less invasive option compared to traditional method that involve jugular vein catheterization which can be complex and risky^10,23^. These methods^10,13,14^ typically involve repetitive, discontinuous exchanges that result in high experimental costs, time, and increased probability of procedural failure. Conversely, our approach permits a long-term observation of treated animals with minimal surgical trauma, thus reducing the surgical trauma and complications previously experienced.

Our study using male rats in this experiment was aimed to reduce the potential physiological data variability caused by gender differences. A successful catheterization of the leg artery or tail vein could be critical for this method. Femoral artery catheterization can be referred to the classic experimental operation video^35^. The femoral artery catheterization can result in relatively small wounds, while also facilitating postoperative animal care compared to the jugular vein catheterization. The tail vein is superficial and conveniently accessible to facilitate implement catheterization. Experimental animals can recover rapidly post-procedure without an additional skin incision that may lead to undue injury.

The continuous blood exchange process must be conducted with catheters free of air bubbles to prevent experimental animals from dying from air embolisms. The balance speed of fluid inflow and outflow is vital for success, we recommend that all fluid flow rates should be between 200 and 300 μL/min for this experiment as the excessive speed may cause red blood cell damage in the blood, while slow speed may cause catheter blockage, both of which can lead to experimental failure.

There are some critical aspects of this study design that must be emphasized: isoflurane induction concentrations must keeping the concentration at 3–4% and maintaining it at 1.5–2%, with a flow rate of 0.2 L per minute, is recommended to ensure that the experiment proceeds smoothly^36–38^. Please ensure the stability of the physiological status of the experimental animals during anesthesia, which will directly affect the difficulty of vascular cannulation. An animal temperature maintenance instrument should be used to ensure the B.T. of the rats. To prevent experimental failure caused by vascular leakage after surgery, please ensure that the vascular ligation is successful as well.

The implementation of a DIHA model followed by WBE intervention led to notable enhancements in hematological metrics, including elevated RBC and HGB levels, as well as reduced WBC and NEUT counts. These alterations were concomitant with observable improvements in the rats' paw condition, suggesting enhanced peripheral blood circulation. The results of the study illustrate the effectiveness of WBE therapy in mitigating DIHA in rats, highlighting its promise as a therapeutic approach. Meanwhile, the rats showed a recovery of hematological metrics by Day 7, indicating WBE treatment permits a long-term observation of treated animals with minimal surgical trauma for intended study.

Nevertheless, some limitations remain in this study. Achieving proficiency in arterial cannulation is critical to minimize animal sacrifice and ensure the success of the procedure, which required practice more from the experimental technician. Although our WBE method shows promising results in rats, the equipment used in our method need to be further adjusted, which could be adaptt to WBE of mice. Furthermore, we will increase our sample size to improve the reliability and validity of our results. This expansion is important for enhancing the accuracy of our data and allowing for more detailed subgroup analyses, making our research widely applicable.

This experimental method through femoral artery-tail vein catheterization follows the 3Rs principle, promoting animal welfare provides a clinically relevant and ethically humane method for blood exchange in animal experiments. Catheterization of the femoral artery can result in a wound that can be minimized and ligated, allowing for the separation of the experimental animals from the catheter. Through tail vein catheterization which reduces the need for surgical wounds, minimizing the trauma and pain experienced by the animals during surgery. Our blood exchange procedures not only have the potential to reduce the invasiveness and trauma, but also provide a new research method for animal experiments and ultimately contribute to exploration in biomedical research.

The establishment of a continuous blood exchange model in rats via femoral artery-tail vein cannulation is a significant advancement in animal experimentation research. This refined method offers a clinically relevant approach and simplifies the experimental procedure, reducing the burden on the animals. This novel approach has the potential to provide insight into the efficacy mechanism of blood exchange in treating various disease conditions, thus serving for future studies.

### Supplementary Information

Supplementary Information.

Supplementary Video 1.

## Data Availability

The data supporting the findings of this study are openly available in the research data file and Supplementary Table 2. And the study adheres to ARRIVE Essential 10 of the ARRIVE guidelines 2.0 including compliance in reporting in vivo experiments, animal details, sample size, procedures, randomization, blinding, and statistics, underscores the study's quality, transparency, and scientific applicability.
