# Supplementary material for: Continuous blood exchange in rats as a novel approach for experimental investigation
Source: Sci Rep. 2024 May 28;14:12194. doi: 10.1038/s41598-024-63049-0 (PMC11133302; doi:10.1038/s41598-024-63049-0)
Supplement: Supplementary file 1 — Supplementary Information. [file 41598_2024_63049_MOESM1_ESM.doc]

**Supplementary Table 1.** Comparative Summary of Animal Models for Blood Exchange Procedures

| **References** | **Animal** | **Method** | **Characteristics** |
| --- | --- | --- | --- |
| Visser, K R et al. *Cardiovascular research.* (1990) ^[1]^ | Dog | 100ml blood was withdrawn using the left femoral artery and 100ml hemoglobin solution was infused by femoral vein in each exchange. | Hemoglobin solution, Repeated invasive exchanges, heightened procedural risks, discontinuous blood replacement, High cost of experimental animals. |
| Mongan, Paul D et al. *The Journal of trauma*. (2009) ^[2]^ | Pig | Each animal received three exchange transfusions to gradually reduce their blood volume by 10%, 30%, and 50%, replacing it with either HBOC-201 or human serum albumin in equal amounts. | HBOC-201 or human serum albumin, repetitive invasive exchanges, heightened procedural risks, discontinuous blood replacement, High cost of experimental animals. |
| Zhou, Ning et al. *Journal of hepatology.* (2015) ^[3]^ | Pig | Blood was drawn from the jugular vein at a flow rate of 50 ml/min, filtered through the OP-02W plasma filter, and re-injected into the animal after the PE procedure. | Combined application of coupled low-volume plasma exchange with plasma filtration. Complex, resource-heavy, high cost of experimental animals. |
| Urayama, Akihiko et al. *Molecular psychiatry.* (2022) ^[4]^ | Mice | 300 μL of blood was taken from Tg2576 mice and replaced with 300 μL of blood from wild-type mice once a month for 10-14 months. Replace 40-60% of the original blood each time. | Therapy Alzheimer's disease by whole blood exchange. Long term catheterization, partial blood exchange, lengthy duration, the risk of a blood vessel wall rupture. |
| Pei Siya. et al. *Scientific reports*. (submitted) | Rat | Blood was taken from of DIHA rats and replaced with blood from healthy wild-type rat lasts for 70-100 minutes. Replace over 80-90% of the original blood each time. | Therapy multiple disease which needs whole blood exchange. High efficiency of exchange rate. Continuous single-pass exchange. Surgical simplicity and reproducibility. Ready availability of animal subjects. Minimal invasiveness Low mortality risk. Rapid postoperative animal recovery |

**Supplementary Table 2.** Variations in physiological parameters over time.

| **Metrics** | **Time** | **Control** | **DIHA** | **DIHA +WBE** | **Control vs.**  **DIHA** | **Control vs.**  **DIHA+WBE** |
| --- | --- | --- | --- | --- | --- | --- |
|  |  | **Mean±SD** | **Mean±SD** | **Mean±SD** | **P** | **P** |
| RBC  (10*^12^/L) | Day-1 | 8.1±0.12 | 7.5±0.48 | 7.0±0.75 | 0.3877 | 0.2534 |
|  | Day 0 | 7.8±0.33 | 4.3±0.28 | 4.7±0.19 | 0.0189* | 0.0029** |
|  | Day 1 | 6.9±0.26 | 3.1±0.16 | 5.5±0.50 | 0.0039** | 0.1407 |
|  | Day 3 | 6.5±0.21 | 1.9±0.20 | 5.1±0.11 | 0.0034** | 0.0287* |
|  | Day 5 | 6.1±0.19 | 3.0±0.31 | 5.6±0.28 | 0.0145* | 0.3427 |
|  | Day 7 | 6.6±0.29 | 5.2±0.33 | 5.6±0.08 | 0.0680 | 0.0649 |
| WBC  (10*^9^/L) | Day-1 | 12.3±2.65 | 10.8±3.53 | 9.4±1.32 | 0.8148 | 0.4539 |
|  | Day 0 | 12.6±3.14 | 36.0±4.58 | 35.0±1.36 | <0.0001**** | <0.0001**** |
|  | Day 1 | 12.1±4.25 | 30.8±11.25 | 21.0±5.69 | <0.0001**** | 0.0029** |
|  | Day 3 | 13.0±3.75 | 19.4±5.20 | 14.4±2.90 | 0.0321* | 0.8255 |
|  | Day 5 | 13.5±4.15 | 17.9±3.78 | 11.5±2.10 | 0.1609 | 0.6749 |
|  | Day 7 | 12.1±1.81 | 11.7±2.11 | 10.2±1.24 | 0.9841 | 0.6984 |
| HGB  (g/L) | Day-1 | 151.0±3.61 | 149.3±6.43 | 154.0±12.29 | 0.9403 | 0.9134 |
|  | Day 0 | 154.7±4.73 | 102.3±9.07 | 113.7±3.51 | 0.0143* | 0.0139* |
|  | Day 1 | 145.3±3.51 | 76.7±3.51 | 117.0±6.00 | 0.0005*** | 0.0046** |
|  | Day 3 | 147.0±2.65 | 59.3±3.51 | 105.3±4.93 | 0.0001**** | 0.0197** |
|  | Day 5 | 120.0±1.00 | 85.0±1.00 | 131.7±6.66 | 0.0004*** | 0.1297 |
|  | Day 7 | 131.7±0.58 | 116.3±5.69 | 134.0±1.00 | 0.0881 | 0.0359* |
| NEUT  (10*^9^/L) | Day-1 | 1.0±0.31 | 0.7±0.20 | 0.7±0.28 | 0.9553 | 0.9730 |
|  | Day 0 | 1.7±1.02 | 25.5±4.65 | 27.8±1.76 | <0.0001**** | <0.0001**** |
|  | Day 1 | 1.3±0.48 | 19.2±3.71 | 5.5±2.73 | <0.0001**** | 0.0077** |
|  | Day 3 | 1.4±0.28 | 5.2±2.07 | 3.7±0.11 | 0.0151* | 0.1734 |
|  | Day 5 | 2.2±0.57 | 2.7±0.81 | 2.4±0.62 | 0.9070 | 0.9844 |
|  | Day 7 | 1.5±0.54 | 0.8±0.30 | 1.5±0.60 | 0.8546 | >0.9999 |
| PLT  (10*^9^/L) | Day-1 | 760.0±162.86 | 764.3±80.36 | 948.3±5.51 | 0.9957 | 0.3069 |
|  | Day 0 | 864.0±115.19 | 548.3±95.66 | 920.3±55.9 | 0.2039 | 0.7914 |
|  | Day 1 | 623.7±282.34 | 576.0±143.13 | 543.0±182.56 | 0.8843 | 0.9068 |
|  | Day 3 | 922.3±125.05 | 427.3±179.05 | 681.0±199.18 | 0.0725 | 0.4805 |
|  | Day 5 | 839.7±244.20 | 586.7±161.80 | 661.3±326.00 | 0.5341 | 0.6058 |
|  | Day 7 | 985.7±239.29 | 757.7±266.90 | 725.7±333.10 | 0.1783 | 0.1094 |
| B.W.(g) | Day-1 | 362.5±14.29 | 361.5±8.48 | 359.0±2.69 | 0.9870 | 0.9254 |
|  | Day 0 | 358.1±15.03 | 337.1±8.75 | 346.3±5.31 | 0.2648 | 0.5935 |
|  | Day 1 | 369.3±16.5 | 320.6±9.31 | 343.3±3.00 | 0.0822 | 0.1662 |
|  | Day 3 | 386.2±19.81 | 334.9±8.75 | 354.5±6.60 | 0.1216 | 0.1270 |
|  | Day 5 | 391.2±16.91 | 355.0±7.32 | 367.7±6.36 | 0.0664 | 0.3226 |
|  | Day 7 | 404.0±20.95 | 360.2±10.35 | 375.6±8.21 | 0.1170 | 0.2885 |
| B.T. (℃) | Day-1 | 36.8±0.31 | 37.2±0.06 | 37.3±0.35 | 0.2548 | 0.5259 |
|  | Day 0 | 37.1±0.15 | 37.1±0.44 | 37.0±0.12 | 0.9940 | 0.0681 |
|  | Day 1 | 37.2±0.36 | 37.0±0.35 | 37.0±0.44 | 0.5150 | 0.9050 |
|  | Day 3 | 36.9±0.40 | 36.4±0.17 | 36.6±0.06 | 0.1513 | 0.3579 |
|  | Day 5 | 37.5±0.23 | 36.8±0.15 | 36.9±0.47 | 0.1785 | 0.2618 |
|  | Day 7 | 37.3±0.57 | 37.2±0.38 | 36.6±0.00 | 0.9979 | 0.3066 |

**Supplementary Table 2.** Variations in physiological parameters over time. Mean ± SD of RBC, WBC, HGB, NEUT, PLT, Body weight (B.W.) and Body temperature (B.T.) before and after treatment (each group n=3).
